# Intensive Endoscopic Resection for Downstaging of Superficial Non‐ampullary Duodenal Epithelial Tumor Burden in Familial Adenomatous Polyposis: A Single‐center Retrospective Study on Long‐term Clinical Outcome

**DOI:** 10.1002/deo2.70419

**Published:** 2026-09-11

**Authors:** Reona Kawamura, Masayoshi Yamada, Hiroyuki Takamaru, Takeshi Nakajima, Seiichiro Abe, Satoru Nonaka, Ichiro Oda, Noriko Tanabe, Makoto Hirata, Yutaka Saito

**Affiliations:** ^1^ Endoscopy Division National Cancer Center Hospital Tokyo Japan; ^2^ Department of Genetic Medicine and Services National Cancer Center Hospital Tokyo Japan; ^3^ Department of Clinical Genetic Oncology Osaka International Cancer Institute Osaka Japan; ^4^ Kawasaki Rinko Hospital Kanagawa Japan

**Keywords:** bipolar snare, familial adenomatous polyposis, intensive downstaging polypectomy, single‐center retrospective study, superficial non‐ampullary duodenal epithelial tumors

## Abstract

**Background and Study Aims:**

Familial adenomatous polyposis (FAP) is a genetic condition that increases the risk of developing colorectal polyposis and cancer, as well as superficial non‐ampullary duodenal epithelial tumors (SNADETs). In this single‐center retrospective study, we investigated the long‐term clinical outcomes of intensive endoscopic resection for downstaging the SNADET burden (intensive downstaging polypectomies [IDPs]) in individuals with FAP.

**Methods:**

Of 56 patients with FAP who underwent endoscopic treatment of SNADETs, 34 underwent IDPs, and 22 underwent conventional treatments, with a median follow‐up period of 71 months. IDP was defined as the resection of ≥ 3 lesions in one treatment.

**Results:**

The total number of resected lesions was 495 lesions (9.7 ± 7.5 lesions/treatment [average ± standard deviation]) in the IDP group and 42 (1.4 ± 0.5 lesions/treatment) in the conventional group. No delayed bleeding was observed in the IDP group and one in the conventional group. No perforation was observed in our study. The 5‐year incidence rates of high‐grade dysplasia, invasive cancer, and cause‐specific duodenectomy in the IDP group were 15.6%, 3.6%, and 0%, respectively. Downstaging of the Spigelman stage (SS) was achieved in 50% (17/34) of patients in the IDP group.

**Conclusions:**

This study demonstrated that IDP was safe and effective in downstaging the burden of SNADETs, and no duodenectomy due to SNADETs was required during the available follow‐up period in individuals with FAP, even at SS IV.

**Trial Registration:**

N/A.

## Graphical abstract

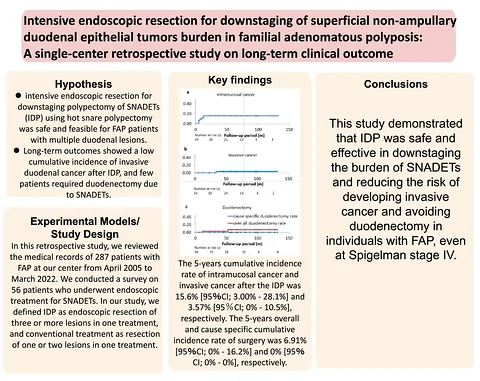

## 1 Background and Aims

Familial adenomatous polyposis (FAP) is a genetic condition that increases the risk of developing colorectal polyposis and cancer, as well as duodenal neoplasms including superficial non‐ampullary duodenal epithelial tumors (SNADETs). Duodenal cancer accounts for 6% of all deaths among FAP patients and is the third major cause of death [1, 2]. The incidence of duodenal cancer increases with age, and the cumulative incidence of duodenal cancer was reported to reach 4.5% at age 57 [3]. The Spigelman classification is widely used to guide surveillance and treatment, and the incidence of duodenal cancer varies according to the Spigelman classification. While the incidence was 0.7% in patients with Spigelman stage (SS) I–III, duodenal cancer occurred in 7%–36% of patients with SS IV in 7.6–10 years [4, 5, 6]. Although endoscopic treatment of duodenal lesions has been performed [7, 8], for FAP patients, the Clinical Practice of Hereditary Colorectal Cancer guidelines 2024 recommend that endoscopic treatment should be considered for FAP patients with SS I–III [9]. On the other hand, a high complication rate and recurrence rate after endoscopic treatment are noted in patients with SS II–III [10], potentially making management even more challenging. Furthermore, surgery is recommended for patients with SS IV rather than endoscopic treatment [9]. Considering the particular situation in these FAP cases, the primary aim of endoscopic treatment should focus on safer procedures and avoiding duodenectomy in the long term. Reports on the efficacy and safety of endoscopic treatment for FAP are limited; Although intensive downstaging polypectomy (IDP) using cold snare polypectomy (CSP) or cold forceps polypectomy (CFP) has been reported [11], its long‐term efficacy remains unclear.

In this single‐center retrospective study, we investigated the long‐term clinical outcomes of intensive endoscopic resection as a means of downstaging the burden of SNADETs (IDPs) in individuals with FAP.

## 2 Materials and Methods

### 2.1 Patients

**FIGURE 1:**
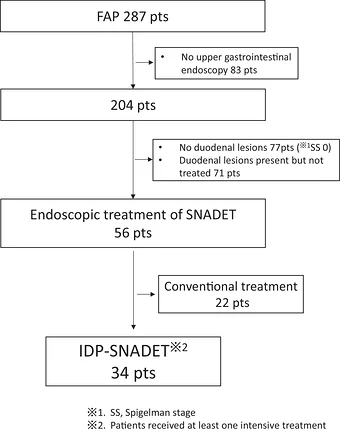
Flow chart of our study.

In this retrospective single‐center observational study, we reviewed the medical records of 287 patients with FAP at the National Cancer Center Hospital, Tokyo, Japan, from April 2005 to March 2022 (Figure 1). After excluding 83 patients who did not undergo upper gastrointestinal endoscopy, 204 patients with FAP underwent upper gastrointestinal endoscopy, and the duodenum was observed. We excluded 77 patients with no duodenal lesions and 71 patients with duodenal lesions who were not endoscopically treated. Finally, we conducted a survey of 56 patients who underwent endoscopic treatment for SNADETs. Among them, we defined IDP as endoscopic resection of three or more lesions in one treatment, and conventional treatment as resection of one or two lesions in one treatment. IDP was performed in 34 patients, and the conventional treatments were performed in 22 patients.

FAP was diagnosed when patients had a history of more than 100 colorectal adenomatous polyps and/or when genetic testing proved the presence of a germline pathogenic variant (GPV) in the *APC* gene (Table S1).

Clinicopathological characteristics of the patients and resected lesions, including age, gender, GPV in the *APC* gene, SS, maximum endoscopic tumor size in diameter of the resected lesions, histopathological tumor grade, and endoscopic treatment outcomes including number of treatments performed, number of resected lesions, number of retrieved lesions, procedure time, number of endoscopic clips for closure, and adverse events, were analyzed. This study was approved by the Ethics Committee of the National Cancer Center, Tokyo, Japan (2013‐303, 2016‐447).

### 2.2 Endoscopic Resection of SNADETs

**FIGURE 2:**
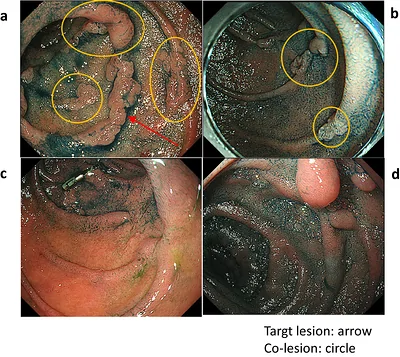
Endoscopic images before and after intensive downstaging polypectomy (IDP). (a, b) Endoscopic images before IDP of a patient with Spigelman stage (SS) IV. (c, d) Endoscopic images after IDP of the same patient as a, b with SS IV.

Indications for duodenal endoscopic resection in FAP patients in the present study were lesions that were sized 15 mm or more in diameter, or biopsy‐proven high‐grade dysplasia or suspicious for adenocarcinoma. At the time of the endoscopic resection for the target lesions, IDP was also performed for other multiple duodenal lesions (Figure 2).

All endoscopic procedures were performed using Olympus gastrointestinal endoscopes (GIF‐Q260J, GIF‐H290T; Olympus, Tokyo, Japan) with carbon dioxide insufflation. An electrosurgical generator (ERBE VIO 300D; Elektromedizin, Tübingen, Germany) was used. A bipolar snare (DORAGONARE; ZEON Medical Inc., Tokyo, Japan) was used for hot snare polypectomy. A distal attachment cap (Elastic Touch; TOP Medical, Tokyo, Japan) was routinely employed during the procedures. The electrosurgical generator was set to FORCED COAG mode, effect 3, with an output of 20 W.

**FIGURE 3:**
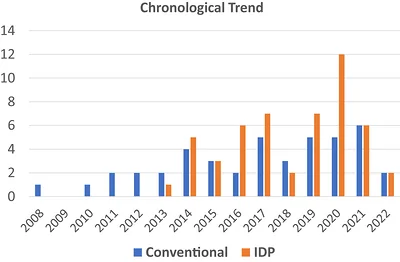
Chronological trend between conventional treatment and intensive downstaging polypectomy (IDP).

There was a chronological trend between both treatment methods (Figure 3), and IDP was introduced in 2013, while conventional treatment had been performed before that. Patients with three or more target lesions are generally included in the IDP group. However, when a large lesion exceeding 30 mm was present, initial treatment was targeted at that lesion, with the remaining lesions treated with IDP in subsequent sessions.

We chose endoscopic mucosal resection (EMR) or piecemeal EMR (EPMR) for the lesions that were 10 mm or more in diameter, and polypectomy for the lesions smaller than 10 mm. Prophylactic clipping after endoscopic treatments was performed as required in polypectomy and was always performed in EMR. A total of seven endoscopists performed the conventional treatments (Masayoshi Yamada, Takeshi Nakajima, Satoru Nonaka, Hiroyuki Takamaru, Seiichiro Abe, Ichiro Oda, and Yutaka Saito), and two endoscopists performed the IDPs (Masayoshi Yamada and Takeshi Nakajima).

The histopathological evaluation methods and follow‐up protocol are described in the Supporting Information Methods.

### 2.3 Study Outcomes

The primary outcome was changes in SS in the IDP group.

Secondary outcomes included clinicopathological differences between those who achieved downstaging and those who did not, cumulative incidence rates of intramucosal cancer, invasive cancer, and duodenectomy in the IDP group, and procedure‐related adverse events.

### 2.4 Confirmation of Clinical Outcome Data and Statistical Analysis

Follow‐up clinical outcome data were collected between February and April 2025. These data included all endoscopic examination data, SS, incidence date of high‐grade dysplasia, invasive cancer, surgery (duodenectomy), and date of death.

All statistical analyses were calculated using BellCurve for Excel version 3.20 (Social Survey Research Information Co., Ltd., Tokyo, Japan). Patients who received at least one IDP were included in the IDP group. Patients who did not receive IDP were included in the conventional group. The baseline characteristics of the IDP and conventional group were compared using Fisher's exact test for binary data and the Mann–Whitney U test for continuous variables. Cumulative incidence rates of high‐grade dysplasia, invasive cancer, and surgery (pancreas‐sparing duodenectomy or pancreatoduodenectomy) were estimated using Kaplan–Meier analysis. A *p*‐value ≤0.05 was considered statistically significant.

## 3 Results

### 3.1 Clinical Characteristics of Patients

Patients’ characteristics are summarized in Table 1. The average age was 46 years. Thirty patients (53%) were male. The average follow‐up period was 71 months, and no significant differences were observed in baseline characteristics between the two groups. The number of resected lesions per treatment was 9.7 ± 7.5 lesions (average ± standard deviation) in the IDP group, whereas it was 1.4 ± 0.5 lesions in the conventional group. Regarding the initial SS, 27% of all patients were classified as SS IV. Patients in the IDP group tended to present with more advanced stages (SS III–IV) compared with those in the conventional group (89% [30/34] vs. 55% [13/22], *p* = 0.012).

**TABLE 1:** Clinicopathological characteristics.

	Total	IDP^※^	Conventional	*p*
No. of patients, *n*	56	34	22	−
Age (years) (average ± standard deviation)	46 ± 15.2	45 ± 15.1	49 ± 12.6	0.629
Male/Female, *n*	30/26	20/14	10/12	0.327
Follow‐up period, months (average ± standard deviation)	71.0 ± 36.2	71.2 ± 33.2	70.5 ± 40.5	0.907
No. of resected lesions, *n* (average ± standard deviation)	6.5 ± 7.2	9.7 ± 7.5	1.4 ± 0.5	∓
Initial SS, *n* (%)				0.012^＃^
I	0	0	0	
II	13 (23%)	4 (12%)	9 (41%)	
III	28 (50%)	18 (53%)	10 (45%)	
IV	15 (27%)	12 (35%)	3 (14%)	
V	0	0	0	

IDP, Intensive endoscopic resection for downstaging polypectomy of SNADETs burden.

^※^ Patients received at least one IDP.

^＃^ *p*‐Value was calculated between Spigelman stage I+II and III+IV.

### 3.2 Genetic Characteristics of Patients

Genetic characteristics of the patients are summarized in Table S1. Among the 56 patients included in the study, 41 patients (73%) had a confirmed GPV in the *APC* gene, whereas the remaining 15 patients (27%) were clinically diagnosed with FAP without genetic confirmation.

### 3.3 Treatment Outcome of SNADETs in FAP Patients

Treatment outcomes are summarized in Table 2. IDP was performed 51 times in 34 patients, and the conventional treatments were performed 31 times in 22 patients. In the IDP group, 24 patients underwent IDP once, eight patients twice, one patient three times, and one patient six times. The total number of resected lesions was markedly higher in the IDP group than in the conventional group (495 vs. 42 lesions).

**TABLE 2:** Treatment outcome of the superficial non‐ampullary duodenal epithelial tumors (SNADETs) in familial adenomatous polyposis (FAP) patients.

	IDP^a^ (34 pts)	Conventional (22 pts)	*p*
No. of endoscopic treatments, *n*	51	31	−
No. of resected lesions, *n*	495	42	−
Treatment strategy			
E(P)MR	45	38	<0.001
Polypectomy	450	4	
No. of retrieved lesions, *n* (%)	405 (82%)	37 (88%)	0.306
Largest tumor size, mm (average ± standard deviation)	15.3 ± 7.3	19.6 ± 10.5	0.067
Procedure time, min (average ± standard deviation)	45.8 ± 20.7	35.0 ± 23.7	0.007
No. of polypectomy sites for clip closure, *n*	85 (17%)	42 (100%)	<0.001
Complications, *n*			
Bleeding	0^＊^	1	−
Perforation	0	0	−
Depth of tumor invasion, *n* (%)			
Adenoma	386 (95.4%)	17 (46%)	
Intramucosal cancer	18 (4.4%)	20 (54%)	
Invasive cancer, *n*	1 (0.2%)	0	

^a^ Resected three or more lesions in one treatment.

^＊^95% CI: 0%–6.9%

Among resected lesions, lesions measuring ≥15 mm accounted for 11% (56/495) in the IDP group and 45% (19/42) in the conventional group, indicating that smaller lesions (<15 mm) were more intensively resected in the IDP group. Most of the lesions (450, 91%) were resected by polypectomy in the IDP group, and most of the lesions (38, 90%) were resected by EMR or piecemeal EMR in the conventional group. The number of retrieved lesions in the IDP group was lower than that of the conventional group (82% vs. 88%, *p* = 0.416). The largest resected lesion size in diameter in one treatment was 15.3 ± 7.3 mm in the IDP group and 19.6 ± 10.5 mm in the conventional group (average ± standard deviation). The procedure time was longer in the IDP group (46 min vs. 35 min, *p* = 0.007), which was considered attributable to the greater number of lesions resected per session. Prophylactic clip closure was performed in 17% (85/495) of polypectomy sites in the IDP group, whereas all polypectomy sites were closed in the conventional group. No delayed bleeding was observed in the IDP group and one in the conventional group. No perforation was observed in the present study.

### 3.4 Downstaging of the SS After the IDP

**FIGURE 4:**
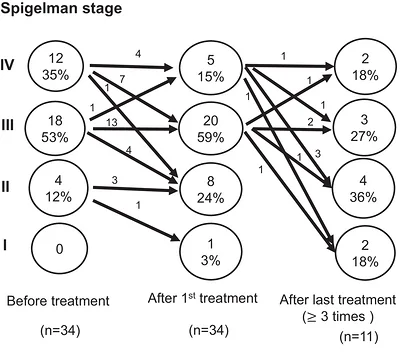
Downstaging of the Spigelman stage (SS) after the intensive downstaging polypectomy (IDP). After a median follow‐up period of 71 months, the SS decreased in 17 patients and remained stable in 17 patients.

All 34 patients underwent at least one follow‐up EGD at one year after the IDP (Figure 4). After the first IDP, the SS decreased in seven patients (21%, 7/34), remained stable in 26 patients (76%, 26/34), and increased in 1 patient (3%, 1/34). After a median follow‐up period of 71 months, the SS decreased in 17 patients (50%, 17/34) and remained stable in 17 patients (50%, 17/34). The average (± standard deviation) change in SS between pre‐treatment and final post‐treatment was 3.2 ± 0.64 and 2.5 ± 0.77 in the IDP group.

### 3.5 Clinicopathological Differences and Treatment Outcome of Those Who Achieved Downstaging and Those Who Did Not in the IDP Group

Of all 34 patients in the IDP group, we compared clinicopathological differences and treatment outcomes between 17 patients who achieved downstaging (downstaging group [DG]) and 17 patients who did not achieve downstaging (non‐DG [NDG]).

Patients’ characteristics are summarized in Table 3. No significant differences were observed in average age, gender, and follow‐up period between the two groups. Regarding the initial SS, the DG tended to have more SS IV patients than the NDG (59% [10/17] vs. 12% [2/17], *p* = 0.013).

**TABLE 3:** Patient characteristics of those who achieved downstaging and those who did not in the intensive downstaging polypectomy (IDP) group.

	DG	NDG	*p*
No. of Patients, *n*	17	17	
Age (years) (average ± standard deviation)	48 ± 21.2	47 ± 16.3	0.88
Male/Female, *n*	8/9	12/6	0.31
Follow‐up period, months (average ± standard deviation)	45 ± 22	44 ± 32	0.91
Initial SS, *n* (%)			0.013^＃^
I	0	0	
II	0	4 (24%)	
III	7 (41%)	11 (65%)	
IV	10 (59%)	2 (12%)	

IDP, Intensive endoscopic resection for downstaging polypectomy of SNADETs burden.

^＃^ *p‐*Value was calculated between Spigelman Stage II + III and IV.

Treatment and pathological outcomes are summarized in Table 4. The number of endoscopic treatments per patient was higher in the DG than in the NDG (2.2 vs. 1.5, *p* = 0.176). The median number of total resected lesions per patient was higher in the DG than in the NDG (10 vs. 6 lesions, *p* = 0.145). The retrieved lesion rate was higher in the NDG than in the DG (90% vs. 77%, *p* < 0.001), which was thought to reflect the number of lesions resected. The mean largest tumor size per session was 15.5 in the DG and 17.7 in the NDG (*p* = 0.46).

**TABLE 4:** Treatment and pathological outcomes of those who achieved downstaging and those who did not in the intensive downstaging polypectomy (IDP) group.

	DG (17 pts)	NDG (17 pts)	*p*
No. of endoscopic treatment/patient, *n* (average ± standard deviation)	2.2 ± 1.8	1.5 ± 0.7	0.176
No. of total resected lesions/patient, *n* (median [IQR])	10 [8–17]	6 [3–15.5]	0.145
No. of retrieved lesions, *n* (%)	238 (77%)	185 (90%)	< 0.001
Largest tumor size, mm (average ± standard deviation)	15.5 ± 7.7	17.7 ± 9.5	0.46
Procedure time, min (average ± standard deviation)	41.8 ± 19.4	39.1 ± 15.3	0.65
No. of polypectomy sites for clip closure, *n*	44 (14%)	41 (20%)	0.08
Complications, n			
Bleeding	0	0	−
Perforation	0	0	−
Depth of tumor invasion, *n* (%)			0.156^a^
Adenoma	224 (94%)	180 (97%)	
Intramucosal cancer	13 (5%)	5 (3%)	
Invasive cancer, *n*	1 (0.4%)	0	

^a^ *p*‐Value was calculated between adenoma and intramucosal cancer/invasive cancer.

### 3.6 Cumulative Risks of Duodenal Neoplasms and Duodenectomy After the IDP

**FIGURE 5:**
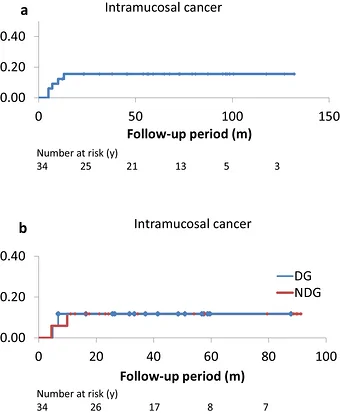
Cumulative incidence rate of high‐grade dysplasia after the intensive downstaging polypectomy (IDP). (a) The 5‐year cumulative incidence rate was 15.6% (95％ CI: 3.0%–28.1%) after a median follow‐up period of 68 months. (b) Comparing downstaging group (DG) and non‐DG (NDG) in the IDP group, the 5‐year cumulative incidence rates were 11.8% (95% CI: 0%–27.8%), respectively.

**FIGURE 6:**
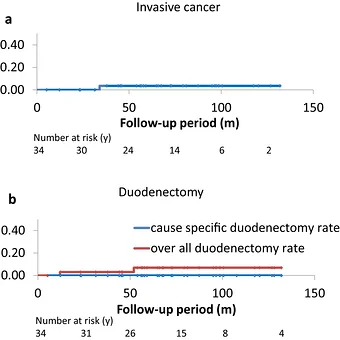
Cumulative incidence rates of invasive cancer and duodenectomy after the intensive downstaging polypectomy (IDP). (a) The rate of invasive cancer. The 5‐year incidence rate was 3.57% (95％ CI: 0%–10.5%) with a median observation period of 66 months. (b) The rate of duodenectomy. The 5‐year overall and cause‐specific cumulative incidence rate of duodenectomy was 6.9% (95% CI: 0%–16.2%) and 0% (95％ CI: 0%–0%), respectively, with a median observation period of 68 months.

After a median follow‐up period of 68 months, 2‐ and 5‐year cumulative incidence rates of high‐grade dysplasia after the IDP were 15.6% (95% confidence interval [95％CI]: 3.00%–28.1%) and 15.6% [95％CI: 3.00%–28.1%] in the IDP, respectively (Figure 5a). When comparing DG and NDG in the IDP group, 2‐ and 5‐year cumulative incidence rates of high‐grade dysplasia were the same, respectively (11.8% [95%CI: 0%–27.8%] and 11.8% [95%CI: 0%–27.8%]) (Figure 5b). The 2‐ and 5‐year cumulative incidence rates of invasive duodenal cancer in the IDP were 0% [95％CI: 0%–0%] and 3.57% [95％CI: 0%–10.5%] (Figure 6a).

During the follow‐up period, no patient underwent surgery due to SNADETs; however, two patients in the IDP group underwent surgery, specifically, pancreas‐sparing duodenectomy for ampullary cancer and pancreaticoduodenectomy for pancreatic cancer, respectively. The 5‐year overall cumulative incidence rate of surgery in the IDP group was 6.91% [95% CI: 0%–16.2%], whereas the 5‐year cause‐specific cumulative incidence rate of duodenectomy due to SNADETs was 0% [95% CI: 0%–0%] (Figure 6b). No surgical interventions were performed in the conventional group.

## 4 Discussion

This study demonstrated that IDP was a safe and effective treatment, and in terms of long‐term outcomes at 71 months, no patients required duodenectomy due to SNADETs.

The IDP group included a significantly higher proportion of patients with advanced stages. Although this difference likely reflects selection bias where IDP was preferentially applied to patients with a larger number of target lesions, we think these outcomes clarify which patients should receive IDP.

Importantly, there were no adverse events after IDP by hot polypectomy, which is comparable to the results of intensive treatment using CSP, in which no cases of post‐bleeding or perforation were reported [11]. Although the clip closure rate in the IDP group was 17%, no delayed bleeding occurred, suggesting that routine clip closure may not be mandatory when the polypectomy ulcer is small. One possible explanation for the low complication rate is the use of a bipolar snare instead of a monopolar snare, as bipolar devices are reported to limit thermal injury to the submucosal and muscular layers [12, 13]. Furthermore, we also think that hot polypectomy allows for more confident resection than CSP in the case that the tumor is an intramucosal or submucosal invasive cancer [12, 14, 15]. In our cohort, one lesion invading 400 µm into the submucosa and venous invasion was found to be positive, but it was endoscopically diagnosed as an adenoma prior to polypectomy. Details of the case have been previously reported [12]. Since it is difficult to endoscopically diagnose adenomas, intramucosal cancer, or submucosal invasive cancer [16], we believe that all resected lesions should be retrieved and submitted for pathological evaluation.

In terms of SS downstaging, SS decreased in 50% of patients in our IDP group, whereas Takeuchi et al. reported SS downstaging in 71% of patients at one‐year follow‐up [11]. This difference may be partly explained by differences in treatment intensity, as fewer lesions were resected per session in our study [11]. In addition, in our study, patients who achieved downstaging tended to have a greater number of lesions resected, suggesting that more extensive lesion clearance may contribute to SS downstaging, particularly in patients with advanced SS. However, direct comparison is difficult because of differences in baseline characteristics, treatment strategy, and follow‐up duration. Although the cumulative incidence of invasive duodenal cancer and duodenectomy was low during the available follow‐up period in both studies, these findings should not be interpreted as evidence of equivalent long‐term disease control. Further follow‐up is needed to determine the optimal extent of lesion clearance.

In the long‐term outcome, only one case of invasive duodenal cancer occurred during the study. The incidence rate of invasive duodenal cancer in the IDP group of our study (3.6% at 5 years after treatment) was comparable to present studies (7% at 7.6 years) [3, 4, 6, 17]. As a result, no cases of SNADET led to duodenal surgical resection, even though this study included 27% of FAP patients with SS IV, for whom surgical intervention is recommended in current guidelines [9]. We believe that this study will provide an opportunity to review the current guidelines about duodenal lesions in FAP patients.

There are some limitations of this study. First, this is a single‐center retrospective study. The number and target lesions to be resected in one treatment were determined by the operator. However, since only seven operators performed SNADET treatment for FAP and only two for IDP in this study, we think the impact of operator bias is relatively minor. Furthermore, a chronological bias existed, as conventional treatment predominated before 2014, whereas IDP became more common thereafter. However, patients requiring three or more treatment sessions were ultimately managed with IDP, minimizing long‐term group overlap. Second, ampullary tumors were not included in this study. Of the 56 FAP patients included in this study, nine underwent papillectomy, and none underwent duodenectomy due to an ampullary tumor. Therefore, we considered that this would not affect the primary outcome, and it was not included in this study. Third, the median follow‐up period was 71 months, and the observation period is relatively short. To establish further data, a longer‐term outcome is needed.

## 5 Conclusion

Intensive endoscopic treatment of SNADETs for FAP patients using hot snare polypectomy can be performed as a safe procedure. IDP was effective for downstaging duodenal tumor burden, and no duodenectomy due to SNADETs was required during the available follow‐up period, even in patients with SS IV. These findings suggest that IDP may be a useful endoscopic management option for selected patients with FAP.

## Author Contributions

**Masayoshi Yamada**: conceptualization, writing – review and editing, and supervision. **Hiroyuki Takamaru**: writing – review and editing. **Takeshi Nakajima**: writing – review and editing. **Yutaka Saito**: writing – review and editing, supervision. **Noriko Tanabe**: writing – review and editing. **Ichiro Oda**: writing – review and editing. **Satoru Nonaka**: writing – review and editing. **Seiichiro Abe**: writing – review and editing. **Makoto Hirata**: writing – review and editing. **Reona Kawamura**: conceptualization, investigation, methodology, visualization, writing – review and editing, and writing – original draft.

## Funding

The authors have nothing to report.

## Ethics Statement

**Approval of the research protocol by an Institutional Reviewer Board**: This study was approved by the Ethics Committee of the National Cancer Center, Tokyo, Japan (2013‐303, 2016‐447).

## Conflicts of Interest

Ms. Noriko Tanabe reports her consulting fees from Konica Minolta REALM and presentation fees from Myriad Genetics G.K, Nippon Kayaku Co., Ltd, Eisai Co., Ltd, MSD G.K., Konica Minolta REALM, and AstraZeneca K.K. Dr. Seiichiro Abe reports his personal fees and research funding from Olympus, Boston Scientific, and FUJIFILM. Dr. Yutaka Saito reports his research funding from Olympus Corporation and paid speaker fees from Olympus Marketing Inc. and Erbe Elektromedizin GmbH.

The requirement for written informed consent was waived because of the retrospective study.

## Supporting information




**FIGURE S1** Correlation between *APC* gene variant and SS. Patients with SS IV who could not be downstaged by IDP have GPV sites at codons 278, 946, 1061, and 1281.

**TABLE S1** List of pathogenic alterations in this study.

**Supporting Information Methods**: Histopathology evaluation and follow‐up protocol.

**Supporting Information Result**: Genetic characteristics of patients.

**Supporting Information Ethics and STROBE Compliance**: Ethics approval, informed consent, and STROBE compliance.

**Supplementary Discussion**: APC GPV and Spigelman Stage.

**Supplementary Reference**: deo270419‐sup‐0007‐SuppMat5.docx
