# Supplementary material for: Intensive Endoscopic Resection for Downstaging of Superficial Non‐ampullary Duodenal Epithelial Tumor Burden in Familial Adenomatous Polyposis: A Single‐center Retrospective Study on Long‐term Clinical Outcome
Source: DEN Open. 2026 Sep 11;7(1):e70419. doi: 10.1002/deo2.70419 (PMC13569982; doi:10.1002/deo2.70419)
Supplement: Supplementary file 2 [file DEO2-7-e70419-s002.docx]

**Supplementary Result - Genetic characteristics of patients**

Regarding the distribution of GPVs, of 41 patients for whom GPV could be identified, 17 had frameshift, 17 had nonsense, 3 had splicing abnormality, 3 had exon1B pathogenic variants and 1 had no GPV in *APC* (clinically suspected attenuated FAP). Of the 15 individuals without identified GPVs, 12 had not undergone genetic testing, and for the remaining 3, testing was performed at external institutions with unavailable genetic data. The correlation between *APC* gene variant and SS is summarized in Fig S1. Patients with SS IV have various GPV sites at codon 154, 278, 696, 723, 946, 1061, 1062, 1187, 1281, 1307. Patients with SS IV who could not be downstaged by IDP have GPV sites at codon 278, 946, 1061, 1281.
