# Supplementary material for: Intensive Endoscopic Resection for Downstaging of Superficial Non‐ampullary Duodenal Epithelial Tumor Burden in Familial Adenomatous Polyposis: A Single‐center Retrospective Study on Long‐term Clinical Outcome
Source: DEN Open. 2026 Sep 11;7(1):e70419. doi: 10.1002/deo2.70419 (PMC13569982; doi:10.1002/deo2.70419)
Supplement: Supplementary file 3 [file DEO2-7-e70419-s003.docx]

# Supplementary Methods –Histopathology evaluation and Follow-up Protocol

**Histopathological evaluation:**

Specimens resected by EMR were fixed in formalin and sectioned at 2–3 mm intervals. Specimens resected by polypectomy were sectioned at the maximum diameter. Histological classification was performed according to the World Health Organization. Tumor grade and depth of invasion were classified as low-grade adenoma/dysplasia, high-grade adenoma/dysplasia, and submucosal invasive cancer. The deepest point of submucosal invasion was measured from the lower border of the muscularis mucosae. Curative resection was defined as lesions showing low- or high-grade adenoma/dysplasia regardless of margin status, reflecting the need to minimize mucosal defects in FAP. Lesions with lymphovascular invasion, poorly differentiated components, or submucosal invasion were classified as noncurative.

**Follow-up protocol:**

All patients (IDP and conventional group) underwent intensive follow-up at least one year after endoscopic treatment. Patients with curative resection underwent repeat IDP until no lesions ≥15 mm remained. Patients with noncurative resection were recommended to undergo additional surgery; if declined, they were followed with CT or abdominal ultrasonography and serum tumor markers (CA19-9, CEA) every 6 months for at least 5 years, along with annual upper gastrointestinal endoscopy.
