# Supplementary material for: Intensive Endoscopic Resection for Downstaging of Superficial Non‐ampullary Duodenal Epithelial Tumor Burden in Familial Adenomatous Polyposis: A Single‐center Retrospective Study on Long‐term Clinical Outcome
Source: DEN Open. 2026 Sep 11;7(1):e70419. doi: 10.1002/deo2.70419 (PMC13569982; doi:10.1002/deo2.70419)
Supplement: Supplementary file 4 [file DEO2-7-e70419-s004.docx]

**Supplementary Table1**. List of pathogenic alterations in this study

| Pathogenic  Alterations | Variant  Information | Amino acid  Change | Colorectal  polyposis | SS | No. of Patients |
| --- | --- | --- | --- | --- | --- |
|  |  |  |  |  |  |
| Nonsense | c.477C>G | p.Tyr159X | Sparse | III | 1 |
|  | c.460G>T | p.E154X | Sparse,  Sparse | III, IV | 2^§^ |
|  | c.832C>T | p.Q278X | Profuse,  Profuse | IV, IV | 2^§^ |
|  | c.849C>T | p.Arg283X | Sparse | III | 1 |
|  | c.1381G<T | p.R461X | Profuse,  Sparse | II, III | 2^§^ |
|  | c.1495C>T | p.R499X | Profuse | II | 1 |
|  | c.1495C>T | p.R499X | Profuse | III | 1 |
|  | c.2086G>T | p.Glu696 | Sparse | IV | 1 |
|  | c.4348C>T | p.R1450X | Profuse | III | 1 |
|  | c.2677G>T | p.E893X | Profuse | III | 1 |
|  | c.3842C>A | p.S1281X | Profuse,  Sparse | II, IV | 2^§^ |
|  | c.2079A>T | p.Lys693X | Profuse | III | 1 |
|  | c.2626C>T | p.R876X | Profuse | III | 1 |
| Frameshift | c.1489 | p.L497X | Sparse | III | 1 |
|  | c.1415 | p.497X | Profuse | III | 1 |
|  | c.1863_1866 | p.628X | Profuse | III | 1 |
|  | c.3199_3202 | p.Q1067fs | Profuse | III | 1 |
|  | c.3183_3187 | p.1061fsX2 | Profuse | IV | 1 |
|  | c.3631_3632 | p.1215X | Profuse | II | 1 |
|  | c.3927_3931del | p.Glu1309Aspfs*4 | Profuse | IV | 1 |
|  | c.3475 | p.T1160Nfs*4 | Sparse | III | 1 |
|  | c.2332 | p.N778Ifs*2 | Sparse | II | 1 |
|  | c.4510_4511 | p.S1504fs | Sparse | II | 1 |
|  | c.3922_3925 | p.K1308fs | Profuse | II | 1 |
|  | c.2169 | p.Ala723fsX4 | Profuse,  Profuse | III, IV | 2^§^ |
|  | c.3558 | p.Ile1187TyrfsX21 | Sparse | IV | 1 |
|  | c.2838_2839 | p.T946fs | Sparse | IV | 1 |
|  | c.3183_3187 | p.1062X | Profuse | IV | 1 |
|  | c.3921_3925 | p.I1307fsX4 | Profuse | IV | 1 |
| Splice site | IVS11+1G>T | Splicing | Sparse | II | 1 |
|  | c.1409-1G>A | Splicing | Profuse | III | 1 |
|  | c.532-2A>G | Splicing | Profuse | IV | 1 |
| Exon 1B | chr5:112043162_112050797 | Large scale  Deletion | Profuse | III | 1 |
|  | c.-190G>A |  | Profuse | II | 1 |
|  | c.-190G>A |  | Sparse | III | 1 |

^§^They are parents and children.
