# Supplementary material for: Intensive Endoscopic Resection for Downstaging of Superficial Non‐ampullary Duodenal Epithelial Tumor Burden in Familial Adenomatous Polyposis: A Single‐center Retrospective Study on Long‐term Clinical Outcome
Source: DEN Open. 2026 Sep 11;7(1):e70419. doi: 10.1002/deo2.70419 (PMC13569982; doi:10.1002/deo2.70419)
Supplement: Supplementary file 5 [file DEO2-7-e70419-s005.docx]

**Supplementary Reference**

18, Bjork J, Akerbrant H, Iselius L et al. Periampullary Adenomas and Adenocarcinomas in Familial Adenomatous Polyposis: Cumulative Risks and APC Gene Mutations. Gastroenterology. 2001; 121: 1127-1135

19, Groves C, Lamlum H, Crabtree M et al. Cluster Region, Association Between Germline and Somatic Mutations and Genotype-Phenotype Correlation in Upper Gastrointestinal Familial Adenomatous Polyposis. Am J Pathol. 2002; 160: 2055-2061

20, Bertario L, Russo A, Sala P et al. Multiple Approach to the Exploration of Genotype-Phenotype Correlations in Familial Adenomatous Polyposis. J Clin Oncol. 2003; 21: 1698-1707
