# Supplementary material for: Intensive Endoscopic Resection for Downstaging of Superficial Non‐ampullary Duodenal Epithelial Tumor Burden in Familial Adenomatous Polyposis: A Single‐center Retrospective Study on Long‐term Clinical Outcome
Source: DEN Open. 2026 Sep 11;7(1):e70419. doi: 10.1002/deo2.70419 (PMC13569982; doi:10.1002/deo2.70419)
Supplement: Supplementary file 6 [file DEO2-7-e70419-s006.docx]

# Supplementary Discussion – APC GPV and Spigelman Stage

In the correlation with germline pathogenic variants (GPVs) in familial adenomatous polyposis (FAP) and Spigelman stage, patients with Spigelman stage IV who could not be downstaged by intensive endoscopic downstaging polypectomy (IDP) had GPV sites at codons 278, 946, 1061, and 1281. In previous studies, mutations located downstream from codon 976 were reported to be associated with advanced duodenal disease and Spigelman stage IV [17][18][19]. In the present study, GPVs at codons 1061 and 1281, which are located downstream from codon 976, were also associated with poor response to IDP, consistent with these previous reports. By contrast, poor response in patients with GPVs at codons 278 and 946, which are located upstream from codon 976, has not been previously reported. These codon sites may warrant further investigation as potential predictors of response to intensive endoscopic downstaging.
