# Supplementary material for: Intensive Endoscopic Resection for Downstaging of Superficial Non‐ampullary Duodenal Epithelial Tumor Burden in Familial Adenomatous Polyposis: A Single‐center Retrospective Study on Long‐term Clinical Outcome
Source: DEN Open. 2026 Sep 11;7(1):e70419. doi: 10.1002/deo2.70419 (PMC13569982; doi:10.1002/deo2.70419)
Supplement: Supplementary file 7 [file DEO2-7-e70419-s007.docx]

# Supplementary Methods – Ethics and STROBE Compliance

Ethics approval:
This retrospective observational study was approved by the Ethics Committee of the National Cancer Center, Tokyo, Japan (Approval numbers: 2013-303 and 2016-447).

Informed consent:
Written informed consent was obtained from all patients included in the study. The study was conducted in accordance with the ethical principles of the Declaration of Helsinki.

STROBE compliance:
This study was designed and reported in accordance with the Strengthening the Reporting of Observational Studies in Epidemiology (STROBE) Statement for cohort studies. A completed STROBE checklist has been provided as Supplementary File A to ensure transparent and comprehensive reporting of study design, conduct, analysis, and interpretation.
